# High-Arctic family planning: earlier spring onset advances age at first reproduction in barnacle geese

**DOI:** 10.1098/rsbl.2020.0075

**Published:** 2020-04-08

**Authors:** Mari Aas Fjelldal, Kate Layton-Matthews, Aline Magdalena Lee, Vidar Grøtan, Maarten J. J. E. Loonen, Brage Bremset Hansen

**Affiliations:** 1 Centre for Biodiversity Dynamics, Department of Biology, Norwegian University of Science and Technology (NTNU), 7491 Trondheim, Norway; 2 Arctic Centre, University of Groningen, 9700 AB Groningen, The Netherlands

**Keywords:** population ecology, age at first reproduction, Arctic, climate change, multi-event, state uncertainty

## Abstract

Quantifying how key life-history traits respond to climatic change is fundamental in understanding and predicting long-term population prospects*.* Age at first reproduction (AFR), which affects fitness and population dynamics, may be influenced by environmental stochasticity but has rarely been directly linked to climate change*.* Here, we use a case study from the highly seasonal and stochastic environment in High-Arctic Svalbard, with strong temporal trends in breeding conditions, to test whether rapid climate warming may induce changes in AFR in barnacle geese, *Branta leucopsis*. Using long-term mark–recapture and reproductive data (1991–2017)*,* we developed a multi-event model to estimate individual AFR (i.e. when goslings are produced). The annual probability of reproducing for the first time was negatively affected by population density but only for 2 year olds, the earliest age of maturity. Furthermore, advanced spring onset (SO) positively influenced the probability of reproducing and even more strongly the probability of reproducing for the first time. Thus, because climate warming has advanced SO by two weeks, this likely led to an earlier AFR by more than doubling the probability of reproducing at 2 years of age. This may, in turn, impact important life-history trade-offs and long-term population trajectories.

## 1. Introduction

Global warming may have dramatic eco-evolutionary consequences [1,2] by changing long-term population dynamics [3] and the evolution of life-history traits [4,5]. The fastest warming occurs in the Arctic [6], where, as a consequence, the timing of snow melt and vegetation growth onset in spring is advancing rapidly [7,8]. Since the snow-free season is extremely short at high latitudes, prolonged snow cover often has detrimental effects on reproduction in ground-nesting birds [9]. Accordingly, advancing springs due to recent climate warming have proven beneficial [3,10]. Changes in age-specific breeding success can trigger changes in key life-history traits like the age at which individuals mature [11] or reproduce [12] for the first time. Age at first reproduction (AFR) is linked to the fast–slow life-history continuum, where longer-lived species generally exhibit delayed, and larger individual variation in, AFR [13,14]. An individual's AFR will affect its fitness, owing to costs and benefits associated with different life-history strategies [14,15]. Earlier AFR can be beneficial, by increasing the total number of reproductive events, but can come at a cost if resources are used that would otherwise be allocated to growth, survival or future reproduction. Environmental stochasticity and density dependence can also induce variability in AFR [16,17], as high resource competition or poor breeding conditions can lead to individuals delaying maturation [18] or reproduction [11]. While weather conditions are known to influence annual AFR in some species (e.g. common tern, *Sterna hirundo* [19], red deer, *Cervus elaphus* [20]), the link between long-term climate change and trends in AFR remains largely unexplored (but see [21,22]).

Geese migrating to Arctic breeding grounds experience highly variable spring conditions. Consequently, their reproductive success exhibits large inter-annual fluctuations, while adult survival is generally high and buffered against variability [23,24], a common pattern in long-lived species. In Arctic geese, there is substantial age-related variation in reproduction [25], as well as temporal variation associated with timing of nesting, density dependence and food availability [26–28]. Although temporal variation in their AFR has been documented [29,30], potential environmental causes of this variation have received little attention. Accurately estimating AFR can be challenging owing to detection issues and because an individual's breeding state is not always ascertainable. Multi-event models are widely used to quantify state uncertainty, such as mortality [31] or breeding status [32], by evaluating them as a hidden Markov process [33]. Here, using a multi-event framework, we studied causes of variation in AFR, defined as the first production of goslings, in the female portion of a population of Svalbard barnacle geese, *Branta leucopsis*. We hypothesize that an early spring, which has proven beneficial for reproduction overall in this population [34], reduces individual AFR. Since spring onset (SO) is advancing rapidly, this predicts, in turn, a temporal decline in AFR.

## 2. Material and methods

### (a) Study species and data collection

Our study population of breeding barnacle geese is located around Ny-Ålesund (Kongsfjorden), Svalbard (78.9° N, 11.9° E). The Svalbard flyway population overwinters at Solway Firth, UK (55° N, 3.30° W), then travels north in spring with a stopover along mainland Norway before arriving at the Svalbard breeding grounds. Barnacle geese are long-lived (up to 28 years-old) and become sexually mature at 2 years of age [25,35]. They are partial capital breeders, using reserves acquired at wintering and stopover sites earlier in the annual cycle to initiate reproduction [36,37]. Over a 26 year period (1991–2017), 480 female goslings were caught at Ny-Ålesund and ringed with unique colour and metal bands during moulting (July/August). Geese nest on islands during May–June. After hatching, families return to Ny-Ålesund to forage, where ringed adults and associated goslings are recorded, resulting in 3006 individual observations used to model AFR (electronic supplementary material, appendix S1a). Males were excluded from the dataset owing to lower recapture rates [35]. Date of SO and adult population density (POP) were included as time-varying covariates. Accumulated winter snowfall [38] was included initially, but showed no evidence of an effect. SO is the (ordinal) day when the 10 day smoothed daily temperature crosses 0°C and remains above for at least 10 days [39] and has been shown to affect egg production [34]. POP is an annual estimate of adult numbers in the study population, which negatively affects gosling production and fledgling recruitment [34,40].

### (b) Statistical analysis

**Figure 1.**
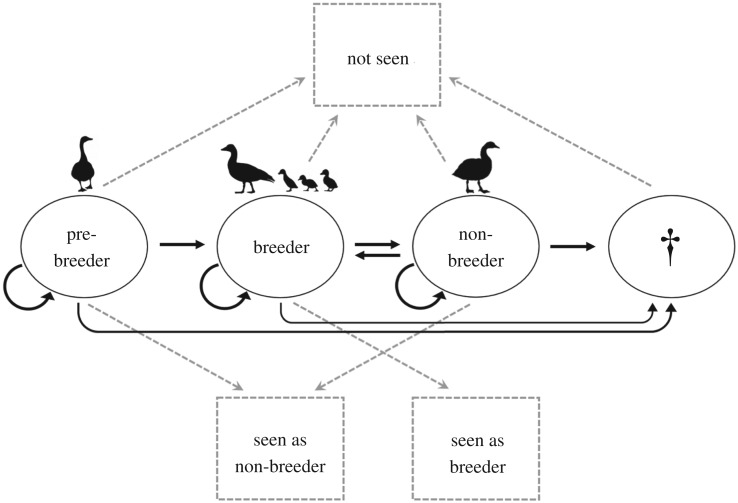
Multi-state model of barnacle geese. Circles represent ‘true', unobservable states, with black arrows indicating transitions between states from time *t*−1 to *t*. Squares are observable events and grey-dotted arrows show which event(s) would be observed given an individual's state.

Mark–recapture data were used to estimate AFR, where reproduction is defined as a female producing goslings (recorded at the foraging grounds, see electronic supplementary material, appendix S1a). Data consisted of individual capture histories of female barnacle geese, recorded as observed with at least one gosling, observed without goslings, or not observed, in a given year. A multi-event model, run in program E-SURGE (Multi-Event SURvival Generalized Estimation; v. 2.1.4 [41]), was used to separate *states*, representing the ‘true' reproductive status of an individual in a given year, and *events*, i.e. the observed state of an individual. We modelled four *states*, pre-breeder (PB), non-breeder (NB), breeder (B) and dead (†). PB was any individual not breeding at year *t* that had never bred previously. NB included individuals not breeding at year *t* but that had bred in a previous year. B was any female that produced at least one gosling at year *t* and † includes dead and permanently emigrated individuals. Three *events* were considered: ‘not seen', ‘seen as breeder' and ‘seen as non-breeder'. Only individuals in the B state could give rise to a ‘seen as breeder' event, whereas both PB and NB states contributed to ‘seen but not breeding' events, and individuals in all three states could be recorded in a ‘not seen' event (figure 1). See table 1 for definitions.

**Table 1.** Explanations of terminology.

terminology	meaning	definition
AFR	age at first reproduction	the age at which a female first produces goslings that survive to the foraging area (around Ny-Ålesund)
state	true annual state	PB, B, NB and †; not always observable; an individual without goslings may be PB or NB, depending on its reproduction history
transition	shift between states from year *t*−1 to year *t*	transition probability from any (living) state at *t*-1 (i.e. B, PB, NB) to state B at year *t* represents the breeding probability at year *t*
event	annual observed reproductive situation	events include seen as a breeder (i.e. with goslings), non-breeder and not observed
PB	pre-breeder	state of females that have yet to produce goslings for the first time (*Note: reproduction probability of PB refers to individuals in PB at t−1 that transitioned into B at t.*)
B	breeder	state of birds producing one or more goslings in a given year
NB	non-breeder	state of birds not producing goslings during breeding season but having bred previously
†	dead	state dead includes dead and permanently emigrated individuals
SO	spring onset date	(ordinal) day when 10 day smoothed daily temperature crosses 0°C and remains above for at least 10 days
POP	population density	annual estimated number of adults in the study population at Ny-Ålesund

Goodness-of-fit (GOF) tests on a simplified, multi-state dataset (*n* = 687, four states: PB, B, NB, not observed) in program U-CARE (v. 2.3.4 [41]) indicated transience, which was accounted for by modelling age-dependent apparent survival, and trap-history-dependent recapture, which was not considered problematic for this analysis (see electronic supplementary material, appendix S1b for details). Details on model implementation are to be found in electronic supplementary material, appendix S1c.

Following [40] and the GOF tests, annual survival probabilities were modelled for goslings, yearlings and adults, including year effects, and recapture probabilities were modelled as year-specific. Transition probabilities (*ψ*) to the breeding state were assumed to be the same from NB and B states (*ψ*^NB/B → B^). We compared models with covariates (SO, POP) on transition probabilities from PB to B (*ψ*^PB → B^) and from NB and B to B (*ψ*^NB/B → B^). An age effect was included on *ψ*^PB → B^, where females of 4 years or older were pooled because of reduced sample sizes thereafter. Model selection was based on Akaike's information criterion corrected for small sample sizes (AICc). A model was considered a better fit when ΔAICc was reduced by at least 2 [42]. Confidence intervals for parameter estimates were calculated using the delta method [43].

Using the Viterbi algorithm in E-SURGE, we reconstituted the 30 most-probable life histories for each individual, and their probabilities, based on the highest-ranked model. From the output, we estimated the AFR distribution in the population and the annual proportion of breeding 2 year olds (electronic supplementary material, appendix S2).

## 3. Results

**Figure 2.**
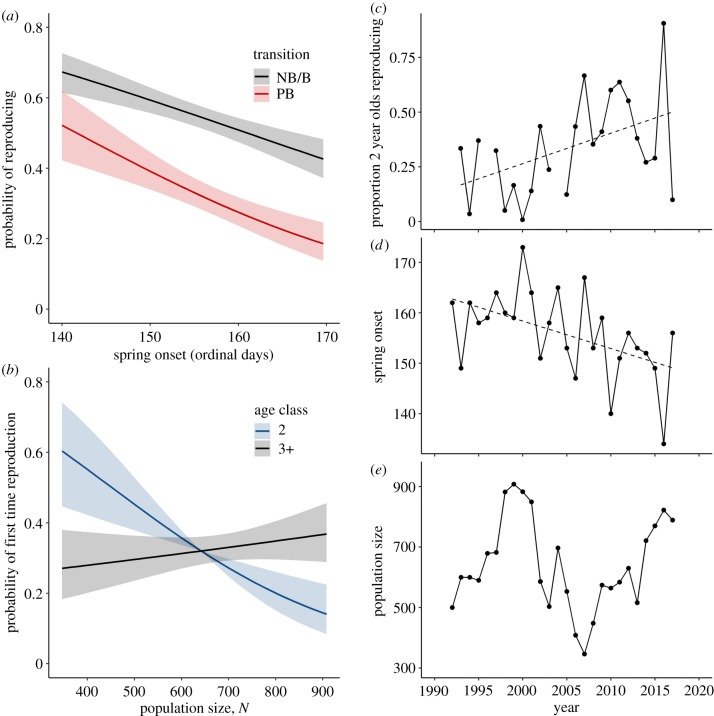
(*a*) Effect of spring onset date, SO, on reproduction probability of first-time (pre-breeders, PB) and experienced (non-breeders or breeders, NB/B) mothers. (*b*) Population density, POP, effects on age classes 2 and 3+ in PB. Annual (*c*) estimated proportion of 2 year olds reproducing, (*d*) SO and (*e*) POP. Dashed lines indicate (*c*) trend towards an increasing proportion of 2 year old individuals reproducing for the first time, estimated with E-SURGE (see Methods), and (*d*) advancing spring phenology.

The best-fitting model (table 2) explaining the pre-breeder to breeder transition (*ψ*^PB → B^) included an effect of SO and an interaction effect between age class and POP. The non-breeder/breeder to breeder transition (*ψ*^NB/B → B^) also included a SO effect (*ψ*^NB/B → B^ (logit scale) *β* = −0.29; 95% CI = −0.40, −0.17), which was weaker than on *ψ*^PB → B^ (−0.44; −0.63, −0.25), as the mean estimate of *ψ*^PB → B^ was outside the confidence interval of *ψ*^NB/B → B^. In other words, the probability of producing goslings decreased with delayed SO and more so for first-time breeders (figure 2*a*). POP had a negative effect on the probability of reproducing for the first time for females of age 2 years (−0.60; −0.93, −0.28) but no effect on ages 3 years and older (0.12; −0.09, 0.34) (figure 2*b*).

**Table 2.** Ten highest-ranked models of transition probabilities for PB and NB/B to B. *k* = number of parameters for transition estimations, excluding survival and recapture (*k* = 54).

rank	model *ψ*^PB → B^	model *ψ*^B/NB → B^	*k*	AICc	ΔAICc
1	age_2–3+_ × POP + SO	SO	6	9760.9	0
2	age_2–3+_ × POP + SO	SO + POP	7	9762.1	1.2
3	age_2–4+_ × POP + SO	SO	7	9762.4	1.5
4	age_2–3+_ × POP age_2–4+_ × SO	SO	9	9763.4	2.5
5	age_2–4+_ × POP + SO	SO + POP	8	9763.5	2.6
6	age_2–3+_ × POP age_2–3+_ × SO	SO	8	9764.9	4.0
7	SO	SO	4	9773.3	12.4
8	SO + POP	SO	5	9773.5	12.6
9	age_2–4+_ × SO + POP	SO	7	9774.3	13.4
10	SO	SO + POP	5	9774.6	13.7

Based on estimated individual AFR, 35% of individuals reproduced for the first time as 2 year olds, while 88 and 97% had reproduced by 5 and 10 years of age, respectively (electronic supplementary material, appendix S2). The top-ranked model suggested that a substantial number of individuals that were not observed as 2 year olds were breeding (appendix S2). Furthermore, the estimated proportion of 2 year olds reproducing each year more than doubled over the study (figure 2*c*) and the date of spring onset, SO, advanced by approximately two weeks (*β* = −0.55, s.e. ± 0.19, *p*-value < 0.01, figure 2*d*). This provides support for our prediction of declining AFR over time with advancing spring phenology. Population densities, POP, showed no significant temporal trend (*β* = −0.01, s.e. ± 4.1, *p*-value = 0.99, figure 2*e*).

## 4. Discussion

This long-term study of Svalbard barnacle geese documents empirically the link between global warming and AFR, a key life-history trait. Although some (poor) individuals produce goslings for the first time later in life, AFR appears strongly linked to annual fluctuations in nest-site and resource availability. Earlier SO increased the probability of producing goslings, especially for females reproducing for the first time, suggesting that inexperienced breeders are more affected by environmental variation. Advancing SO, associated with ongoing climate warming, led to an increasing proportion of reproducing 2 year olds (i.e. age of sexual maturity) over the study. Density dependence, also operating through resource availability, only affected the probability of producing goslings for 2 year olds. This suggests that barnacle geese generally start breeding as 2 year olds and only poor conditions–i.e. cold springs or high densities—force them to delay. In such cases, AFR is likely to change over time with long-term trends in breeding conditions.

Spring phenology can affect AFR since it impacts both clutch success/size and hatching success, through effects on the timing of nesting and food availability during incubation [26,27,34]. Colder springs delay snowmelt, and therefore nest-site availability, but also the timing of food availability by delaying plant growth onset [34]. Similarly, under delayed snowmelt, female geese initially use retained reserves for self-maintenance rather than egg production [44] and take more frequent, and longer, breaks from incubation to forage, increasing egg predation risk [28,45].

Density dependence affects reproduction and thereby potentially the age at which females produce goslings. Here, 2 year olds were less likely to produce goslings in years with higher densities (i.e. higher intraspecific competition), supported by similar findings from a Baltic population of barnacle geese [30]. Reproductive success was also found to be age-dependent in the Baltic population [25], explained by increasing experience/social status with age. This may explain the impact of increased competition on young geese that are forced to settle at sub-optimal nesting sites as densities increase [46]. Better nest-sites have more forage available, limiting time spent off the nest for incubating females, limiting egg predation risk. The same mechanism may also have contributed to stronger effects of SO on pre-breeders (typically younger individuals), since late springs increase snow cover and thereby nest-site availability.

Global warming is having profound effects on reproduction in Arctic geese and other Arctic herbivores [34,47]. Our results, from one of the most rapidly warming places on Earth [6], indicate that climate change is affecting key life-history traits like AFR. Climate change is advancing spring, providing an explanation for the increasing proportion of 2 year olds reproducing and thereby earlier AFR. Reproduction is the main driver of population dynamics in geese, and any changes have substantial population-level effects [40]. However, increased production of goslings will, to some extent, shift the age structure towards a larger proportion of young individuals that are more sensitive to density-dependent processes, potentially counteracting benefits of earlier AFR somewhat. Additionally, here, AFR refers to production of goslings, but survival to fledging is highly variable and susceptible to predation [34,48]. Earlier AFR may also incur a cost through reduced future reproduction or survival, which was not possible to test here, but care should be taken when inferring population-dynamic implications. For long-distance migrants like Arctic geese, following the food-peak across migratory sites is an important evolutionary strategy [49,50]. However, they may, eventually, be unable to keep up with fast-changing spring conditions [51], leading to phenological mismatch in food-web interactions [52,53], with potentially negative reproductive consequences [54]. Nevertheless, this population shows no current indication of mismatch effects [34]. On the contrary, Arctic climate change appears to allow higher gosling production and earlier AFR, which may have positive consequences for population persistence.

## Supplementary Material

Additional fieldwork details, GOF tests and E-SURGE implementation

## Supplementary Material

Results from Viterbi algorithm in E-SURGE

## Data accessibility

Data are available from Dryad (https://datadryad.org/stash/share/jVK4KBYR_SWuyvK83KgFY1bWtYvjPRxvRYubSqkbspw) [55].

## References

[1] Parmesan C 2006 Ecological and evolutionary responses to recent climate change. Annu. Rev. Ecol. Evol. Syst. 37, 637–669. (10.1146/annurev.ecolsys.37.091305.110100)

[2] Walther G-R, Post E, Convey P, Menzel A, Parmesan C, Beebee TJ, Fromentin J-M, Hoegh-Guldberg O, Bairlein F 2002 Ecological responses to recent climate change. Nature 416, 389 (10.1038/416389a) 11919621

[3] Post E et al. 2009 Ecological dynamics across the Arctic associated with recent climate change. Science 325, 1355–1358. (10.1126/science.1173113) 19745143

[4] Winkler DW, Dunn PO, McCulloch CE 2002 Predicting the effects of climate change on avian life-history traits. Proc. Natl Acad. Sci. USA 99, 13 595–13 599. (10.1073/pnas.212251999) 12370441 PMC129719

[5] Parmesan C 2007 Influences of species, latitudes and methodologies on estimates of phenological response to global warming. Glob. Change Biol. 13, 1860–1872. (10.1111/j.1365-2486.2007.01404.x)

[6] Serreze MC, Barry RG 2011 Processes and impacts of Arctic amplification: a research synthesis. Global Planet. Change 77, 85–96. (10.1016/j.gloplacha.2011.03.004)

[7] Foster J 1989 The significance of the date of snow disappearance on the Arctic tundra as a possible indicator of climate change. Arct. Alp. Res. 21, 60–70. (10.2307/1551517)

[8] Bjorkman AD, Elmendorf SC, Beamish AL, Vellend M, Henry GH 2015 Contrasting effects of warming and increased snowfall on Arctic tundra plant phenology over the past two decades. Glob. Change Biol. 21, 4651–4661. (10.1111/gcb.13051) 26216538

[9] Meltofte H, Høye TT, Schmidt NM 2008 Effects of food availability, snow and predation on breeding performance of waders at Zackenberg. Adv. Ecol. Res. 40, 325–343. (10.1016/S0065-2504(07)00014-1)

[10] Gareth K 2004 Predicting impacts of Arctic climate change: past lessons and future challenges. Ecol. Res. 19, 65–74. (10.1111/j.1440-1703.2003.00609.x)

[11] Sæther B-E, Heim M 1993 Ecological correlates of individual variation in age at maturity in female moose (*Alces alces*): the effects of environmental variability. J. Anim. Ecol. 62, 482–489. (10.2307/5197)

[12] Martin K 1995 Patterns and mechanisms for age-dependent reproduction and survival in birds. Am. Zool. 35, 340–348. (10.1093/icb/35.4.340)

[13] Charlesworth B 1994 Evolution in age-structured populations. Cambridge, UK: Cambridge University Press.

[14] Stearns SC 1992 The evolution of life histories. Oxford, UK: Oxford University Press.

[15] Cole LC 1954 The population consequences of life history phenomena. Q. Rev. Biol. 29, 103–137. (10.1086/400074) 13177850

[16] Grøtan V, Sæther B-E, Lillegård M, Solberg EJ, Engen S 2009 Geographical variation in the influence of density dependence and climate on the recruitment of Norwegian moose. Oecologia 161, 685–695. (10.1007/s00442-009-1419-5) 19657678

[17] Sand H 1996 Life history patterns in female moose (*Alces alces*): the relationship between age, body size, fecundity and environmental conditions. Oecologia 106, 212–220. (10.1007/BF00328601) 28307646

[18] Boertje RD, Frye GG, Young DD Jr 2019 Lifetime, known-age moose reproduction in a nutritionally stressed population. J. Wildl. Manage. 83, 610–626. (10.1002/jwmg.21613)

[19] Becker PH, Dittmann T, Ludwigs J-D, Limmer B, Ludwig SC, Bauch C, Braasch A, Wendeln H 2008 Timing of initial arrival at the breeding site predicts age at first reproduction in a long-lived migratory bird. Proc. Natl Acad. Sci. USA 105, 12 349–12 352. (10.1073/pnas.0804179105) PMC2527914 18711134

[20] Langvatn R, Albon S, Burkey T, Clutton-Brock T 1996 Climate, plant phenology and variation in age of first reproduction in a temperate herbivore. J. Anim. Ecol. 65, 653–670. (10.2307/5744)

[21] Jonsson N, Jonsson B 2004 Size and age of maturity of Atlantic salmon correlate with the North Atlantic Oscillation Index (NAOI). J. Fish Biol. 64, 241–247. (10.1111/j.1095-8649.2004.00269.x)

[22] Mangel M 1994 Climate change and salmonid life history variation. Deep Sea Res. II 41, 75–106. (10.1016/0967-0645(94)90063-9)

[23] Clausen P, Frederiksen M, Percival S, Anderson G, Denny M 2001 Seasonal and annual survival of East-Atlantic pale-bellied brent geese *Branta hrota* assessed by capture-recapture analysis. Ardea 89, 101–112.

[24] Kery M, Madsen J, Lebreton JD 2006 Survival of Svalbard pink-footed geese *Anser brachyrhynchus* in relation to winter climate, density and land-use. J. Anim. Ecol. 75, 1172–1181. (10.1111/j.1365-2656.2006.01140.x) 16922853

[25] Forslund P, Larsson K 1992 Age-related reproductive success in the barnacle goose. J. Anim. Ecol. 61, 195–204. (10.2307/5522)

[26] Dickey MH, Gauthier G, Cadieuz MC 2008 Climatic effects on the breeding phenology and reproductive success of an arctic-nesting goose species. Glob. Change Biol. 14, 1973–1985. (10.1111/j.1365-2486.2008.01622.x)

[27] Madsen J, Tamstorf M, Klaassen M, Eide N, Glahder C, Rigét F, Nyegaard H, Cottaar F 2007 Effects of snow cover on the timing and success of reproduction in high-Arctic pink-footed geese *Anser brachyrhynchus*. Polar Biol. 30, 1363–1372. (10.1007/s00300-007-0296-9)

[28] Prop J, de Vries J 1993 Impact of snow and food conditions on the reproductive performance of barnacle geese *Branta leucopsis*. Ornis Scand. 24, 110–121. (10.2307/3676360)

[29] Rockwell R, Cooch E, Thompson C, Cooke F 1993 Age and reproductive success in female lesser snow geese: experience, senescence and the cost of philopatry. J. Anim. Ecol. 62, 323–333. (10.2307/5363)

[30] van der Jeugd H, Larsson K 1999 Life-history decisions in a changing environment: a long-term study of a temperate barnacle goose population. PhD thesis, University of Uppsala.

[31] Fernández-Chacón A, Moland E, Espeland SH, Kleiven AR, Olsen EM 2016 Causes of mortality in depleted populations of Atlantic cod estimated from multi-event modelling of mark–recapture and recovery data. Can. J. Fish. Aquat. Sci. 74, 116–126. (10.1139/cjfas-2015-0313)

[32] Cayuela H, Besnard A, Bonnaire E, Perret H, Rivoalen J, Miaud C, Joly P 2014 To breed or not to breed: past reproductive status and environmental cues drive current breeding decisions in a long-lived amphibian. Oecologia 176, 107–116. (10.1007/s00442-014-3003-x) 24996543

[33] Pradel R 2005 Multievent: an extension of multistate capture–recapture models to uncertain states. Biometrics 61, 442–447. (10.1111/j.1541-0420.2005.00318.x) 16011690

[34] Layton-Matthews K, Hansen BB, Grøtan V, Fuglei E, Loonen MJJE 2019 Contrasting consequences of climate change for migratory geese: predation, density dependence and carryover effects offset benefits of high-arctic warming. Glob. Change Biol. 26, 642–657. (10.1111/gcb.14773) 31436007

[35] Black JM, Prop J, Larsson K 2014 Survival and reproduction. In The barnacle goose (ed. Martin J.), pp. 159–172. London, UK: Bloomsbury Publishing.

[36] Hahn S, Loonen MJJE, Klaassen M 2011 The reliance on distant resources for egg formation in high Arctic breeding barnacle geese *Branta leucopsis*. J. Avian Biol. 42, 159–168. (10.1111/j.1600-048X.2010.05189.x)

[37] Jönsson KI 1997 Capital and income breeding as alternative tactics of resource use in reproduction. Oikos 78, 57–66. (10.2307/3545800)

[38] Peeters B et al. 2019 Spatiotemporal patterns of rain-on-snow and basal ice in high Arctic Svalbard: detection of a climate-cryosphere regime shift. Environ. Res. Lett. 14, 015002 (10.1088/1748-9326/aaefb3)

[39] Le Moullec M, Buchwal A, van der Wal R, Sandal L, Hansen BB 2019 Annual ring growth of a widespread high arctic shrub reflects past fluctuations in community-level plant biomass. J. Ecol. 107, 436–451. (10.1111/1365-2745.13036)

[40] Layton-Matthews K, Loonen MJJE, Hansen BB, Saether B-E, Coste CFD, Grøtan V 2019 Density-dependent population dynamics of a high Arctic capital breeder, the barnacle goose. J. Anim. Ecol. 88, 1191–1201. (10.1111/1365-2656.13001) 31032900

[41] Choquet R, Rouan L, Pradel R 2009 Program E-SURGE: a software application for fitting multievent models. In Modeling demographic processes in marked populations (eds Thomson DL, Cooch EG, Conroy MJ), pp. 845–865. New York, NY: Springer.

[42] Burnham KP, Anderson DR 2002 Model selection and multimodel inference: a practical information-theoretic approach. New York, NY: Springer.

[43] Powell LA 2007 Approximating variance of demographic parameters using the delta method: a reference for avian biologists. Condor 109, 949–954. (10.1093/condor/109.4.949)

[44] Ryder JP 1970 A possible factor in the evolution of clutch size in Ross' goose. Wilson Bull. 82, 5–13.

[45] Greve IA, Elvebakk A, Gabrielsen GW 1998 Vegetation exploitation by barnacle geese *Branta leucopsis* during incubation on Svalbard. Polar Res. 17, 1–14. (10.3402/polar.v17i1.6603)

[46] Stahl J, Tolsma PH, Loonen MJJE, Drent RH 2001 Subordinates explore but dominants profit: resource competition in high Arctic barnacle goose flocks. Anim. Behav. 61, 257–264. (10.1006/anbe.2000.1564) 11170715

[47] Nolet BA, Schreven KH, Boom MP, Lameris TK 2019 Contrasting effects of the onset of spring on reproductive success of Arctic-nesting geese. Auk 137, ukz063.

[48] Loonen MJJE, Tombre IM, Mehlum F 1998 Development of an arctic barnacle goose colony: interactions between density and predation. Norsk Polarinst. Skr. 200, 67–80.

[49] Drent RH, Eichhorn G, Flagstad A, Van der Graaf A, Litvin K, Stahl J 2007 Migratory connectivity in Arctic geese: spring stopovers are the weak links in meeting targets for breeding. J. Ornithol. 148, 501–514. (10.1007/s10336-007-0223-4)

[50] Van der Graaf A, Stahl J, Klimkowska A, Bakker JP, Drent RH 2006 Surfing on a green wave – how plant growth drives spring migration in the barnacle goose *Branta leucopsis*. Ardea 94, 567.

[51] Lameris TK, van der Jeugd HP, Eichhorn G, Dokter AM, Bouten W, Boom MP, Litvin KE, Ens BJ, Nolet BA 2018 Arctic geese tune migration to a warming climate but still suffer from a phenological mismatch. Curr. Biol. 28, 2467–2473. (10.1016/j.cub.2018.05.077) 30033332

[52] Clausen KK, Clausen P 2013 Earlier Arctic springs cause phenological mismatch in long-distance migrants. Oecologia 173, 1101–1112. (10.1007/s00442-013-2681-0) 23660701

[53] Doiron M, Gauthier G, Lévesque E 2015 Trophic mismatch and its effects on the growth of young in an Arctic herbivore. Glob. Change Biol. 21, 4364–4376. (10.1111/gcb.13057) 26235037

[54] Lameris TK, Scholten I, Bauer S, Cobben MM, Ens BJ, Nolet BA 2017 Potential for an Arctic-breeding migratory bird to adjust spring migration phenology to Arctic amplification. Glob. Change Biol. 23, 4058–4067. (10.1111/gcb.13684) 28295932

[55] Fjelldal MA, Layton-Matthews K, Lee AM, Grøtan V, Loonen MJJE, Hansen BB 2020 Individual histories of female barnacle geese Dryad Digital Repository. (10.5061/dryad.wdbrv15jz)

