# Supplementary material for: High-Arctic family planning: earlier spring onset advances age at first reproduction in barnacle geese
Source: Biol Lett. 2020 Apr 8;16(4):20200075. doi: 10.1098/rsbl.2020.0075 (PMC7211454; doi:10.1098/rsbl.2020.0075)
Supplement: Additional fieldwork details, GOF tests and E-SURGE implementation [file rsbl20200075supp1.docx]

**Appendix S1.** Additional fieldwork details, GOF tests and E-SURGE implementation

**Appendix S1a.** Further details of data collected and study system.

Each year, geese arrived at the breeding grounds during May to nest on islands in Kongsfjorden. Goslings hatched around 1st July (range = 19^th^ June to 19^th^ July). Straight after, individuals with newly hatched goslings returned to the coastline to forage around the settlement of Ny-Ålesund. From the beginning of June to the end of August, twice-daily observations were made of individuals and their offspring (goslings) in the study area (around Ny-Ålesund), registering the parental ID (i.e., ringed individuals) and their associated number of goslings. Since parents immediately leave the nesting islands after hatching with their offspring and moved to the foraging grounds, we used these observations of families in the foraging area to determine whether a female had produced goslings (i.e., they would be allocated the ‘seen as breeder’ event). On average, goslings were 3.65 (standard deviation = 2.23) days old when first observed. If a female was never observed with goslings during the breeding season then they would be allocated the ‘seen as non-breeder’ event. If a female was never observed during the breeding season a given year then she would be allocated the ‘not seen event’. Recorded breeder events therefore do not capture females that produce eggs but lose their clutch before arriving at the foraging grounds. These females would be allocated the ‘seen as non-breeder’ event, given that none of their produced offspring that survived until the first recordings at the foraging grounds.

**Appendix S1b.** Results from Goodness-of-fit tests

The Goodness-of-fit test showed significance for transience in the data (p < 0.001), meaning that newly recruited individuals were less likely to be recaptured than individuals with an already established capture history. Because all individuals were marked as goslings in this study, this indicated a difference in apparent survival rates between goslings and adults, as new individuals (goslings) were less likely to be recaptured than individuals with an established recapture history (adults). This was accounted for in the models by adding age-dependent apparent survival. The Goodness-of-fit tests also gave a significant result for trap-history-dependent recapture (p < 0.001). This could be explained by the study system, as barnacle geese showed high fidelity to their breeding and foraging sites. Females using the Ny-Ålesund area for foraging, after leaving the nesting islands, were likely to come back to this site year after year increasing the probability of being recaptured and thus leading to the detection of “trap-happy” individuals. This study consequently analysed data on a non-random sampled population. However, this should not affect the measures of external forces on AFR.

**Appendix S1c.** E-SURGE implementation

Models were implemented in E-SURGE through three main steps: firstly, by constructing matrices for states and events and defining which among-year transitions were possible; an individual with the underlying state PB was restricted from transitioning directly into the NB state, as it needed to previously have bred before it could become a non-breeder. NB and B individuals were restricted from transitioning back to the PB state. The event-matrices defined which underlying states generated observed events; individuals with the states PB or NB were assigned the recorded event “seen but not breeding”, while B individuals was be assigned to the event “seen as successful breeder”. All states could have been assigned the event “not observed”. The matrices were built for estimating survival, transition, and recapture probabilities, and read from rows to columns with each symbol representing the probability for the specific transition. The “-” indicates parameters constrained to a probability of 0. States included in the matrices were; pre-breeder (PB), non-breeder (NB), breeder (B), and “dead” (†). The model construction included five matrices in this setup: one for the initial state; one for survival, defined as the first matrix after the initial state and therefore gave the condition to the following matrices that only surviving individuals could transition between states. The third matrix described transitions between breeding states, where the PB state is prevented from becoming a NB and NB, and B states were prevented from transitioning into the PB state. Fourth, the recapture matrix, defined that individuals from all states could be recorded as “not observed”. Finally, one matrix connected states and events, where it defined that individuals in the PB and NB states will be recorded as non-breeding events if recaptured, while the B state was recorded as a breeding event. The ‘*’ symbol in the matrices indicates redundant entries as each matrix is row-stochastic (the sum of each row sums to 1).

**Initial state matrix**: All the individuals were captured and ringed as goslings, meaning they were all registered as PB at the first occasion.

PB NB B

* − −

**Survival matrix**: Diagonal matrix for survival probabilities.

PB NB B †

PB *y* − − *

NB − *y* − *

B − − *y* *

† − − − *

**Reproduction matrix**: Estimation of reproductive probability, and transition between adult states.

PB NB B †

PB * − *p* −

NB − * *p* −

B − * *p* −

† − − − *

**Recapture matrix:** Recapture probability at the population. NO = not observed, RPB = recaptured pre-breeder, RNB = recaptured non-breeder, RB = recaptured breeder.

NO RPB RNB RB

PB *  *b* − −

NB * − *b* −

B * − − *b*

† * − − −

**Event matrix**: The actual observed reproductive status of an individual was limited to if it is detected with or without goslings (seen as breeder or seen as non-breeder). It was assumed that no observed individual was registered with the wrong event.

Not seen (0) Seen B (1) Seen NB (2)

NO * − −

RPB − − *

RNB − − *

RB − * −

The second main step in the E-SURGE implementation was the model construction, where the models that were to be evaluated through model selection were defined. As an example we present the model construction of the highest ranked model from table 2.

Initial state: i

Transition 1 (survival): a(1,2,3:4)+t

Transition 2 (reproduction): a(1,2:4).f(1).[i+t*x(1)]+a(1,2,3:4).f(1).[t*x(2)]+f(2:3).to(3).[i+t*x(1)]

Recapture: firste+nexte.t

Event: from

In the third step, known probabilities were specified, including the capture probability for the first occasion (i.e. the gosling stage), which was fixed to one, and the breeding probability for yearlings, which was fixed at zero.
