# Supplementary material for: High-Arctic family planning: earlier spring onset advances age at first reproduction in barnacle geese
Source: Biol Lett. 2020 Apr 8;16(4):20200075. doi: 10.1098/rsbl.2020.0075 (PMC7211454; doi:10.1098/rsbl.2020.0075)
Supplement: Results from Viterbi algorithm in E-SURGE [file rsbl20200075supp2.docx]

**Appendix S2.** Results from Viterbi algorithm in E-SURGE

We used the Viterbi algorithm in E-SURGE to reconstitute the 30 most probable life histories for each individual, and their probabilities, based on the highest-ranked model. For most records the cumulative probability of these 30 life histories was close to one (figure S1.1), indicating that this number of life histories was sufficient to capture most of the uncertainty in the estimates. For each record, we calculated the relative probability of each of the 30 suggested life histories and weighted the number of individuals by this probability. For example, if there was a 0.1 probability that an individual with a given record of observations was a breeder at age two, this individual would contribute 0.1 individuals to the count of two-year-old breeders in that year. From the output we estimated the AFR distribution in the population and the annual proportion of breeding two-year-old individuals. Comparing this estimated AFR distribution with the distribution of observed breeding events in the data, we found that E-SURGE estimated a higher rate of breeding in two-year-olds than would be predicted from the observed data alone (figure S1.2). This demonstrates the usefulness of multi-event models in accounting for unobserved events.

**
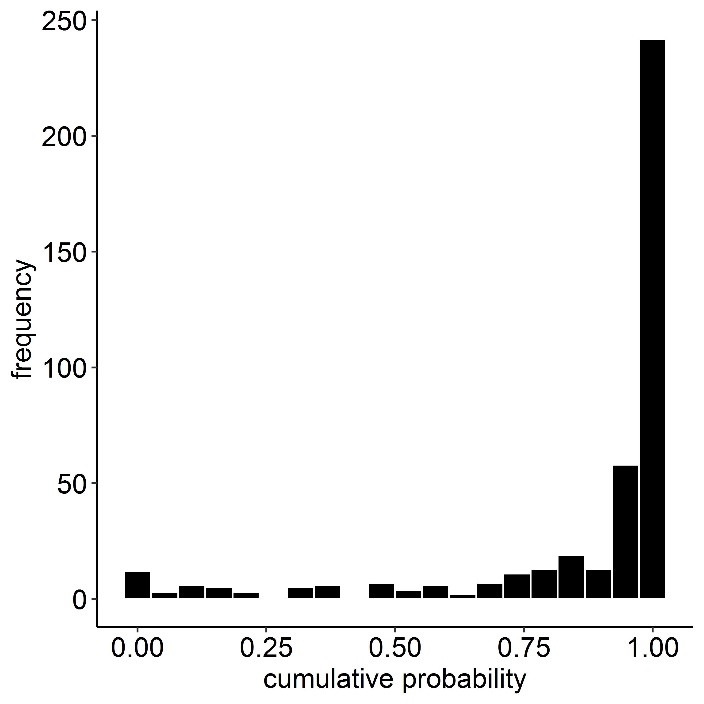
**

**Figure S1.1.** Histogram of the cumulative probabilities of the 30 most probable life histories for each recorded capture history.

**
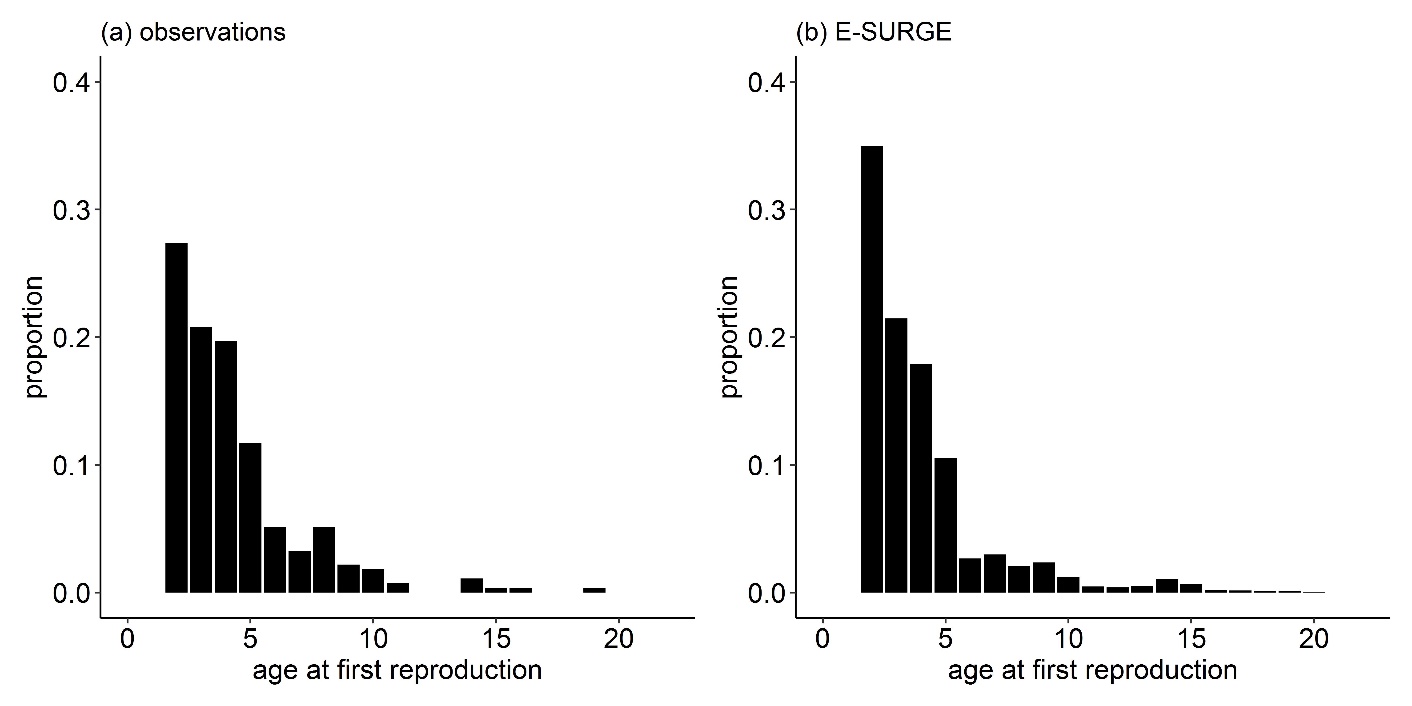
Figure S1.2.** Observed and estimated proportions of ages at first reproduction. Histogram shows the proportions of each age class at which female barnacle geese produced a gosling for the first time (a) based on the observational data (n_individuals,obs_ = 274) and (b) estimated from the highest-ranked model using the Viterbi algorithm in E-SURGE, which estimated a larger proportion of 2-year-old individuals reproducing than were recorded in the data (n_individuals,est_ = 362). NB: estimated proportions for the highest age classes (i.e., from 15 and above) are so low that they involve fewer than one individual in the data set starting to breed, and fewer than five individuals from ages 10 to 15.
